# Epidemiology of Myasthenia Gravis in Sweden 2006–2016

**DOI:** 10.1002/brb3.1819

**Published:** 2020-09-01

**Authors:** Elisabet Westerberg, Anna Rostedt Punga

**Affiliations:** ^1^ Department of Neuroscience Clinical Neurophysiology Uppsala University Uppsala Sweden

**Keywords:** Myasthenia Gravis, neuroepidemiology, Sweden

## Abstract

**Introduction:**

Reported incidence and prevalence rates of Myasthenia Gravis (MG) vary widely and are assumed to have increased over the last few decades. We conducted a nationwide register‐based study on the current incidence and prevalence of MG and MG subgroups in Sweden.

**Methods:**

Data were acquired from four Swedish Health Registers in order to identify patients with MG. Incidence and prevalence rates were calculated for the years 2006–2016, using population numbers provided by Statistics Sweden.

**Results:**

In 2016, the incidence of MG in Sweden was 2.9 per 100,000 inhabitants (95% CI: 2.5–3.2/100,000) and the crude prevalence was 36.1 per 100,000 inhabitants (95% CI: 34.9–37.3). There was a significant increase in Myasthenia Gravis prevalence from 2006 to 2016. Prevalence rates of all MG subgroups but thymoma‐associated MG increased over the same period of time.

**Conclusions:**

The incidence and prevalence of Myasthenia Gravis have increased over time in Sweden, and the rates are high in comparison with other countries.

## Graphical abstract

The epidemiology of Myasthenia Gravis in Sweden was explored in a register‐based setting over the time period 2006–2016. The incidence and prevalence of Myasthenia Gravis in Sweden have increased over time, and the rates are high in comparison with many other countries.

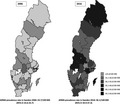

## 1 INTRODUCTION

Myasthenia Gravis (MG) is an autoimmune neuromuscular disease characterized by skeletal muscle weakness and fatigability. Reported incidence and prevalence rates of MG vary widely, with reported yearly incidence rates ranging from 0.2 to 3 per 100,000 inhabitants and prevalence rates ranging from 1.5 to 24 per 100,000 inhabitants (Carr, Cardwell, McCarron, & McConville, 2010; McGrogan, Sneddon, & de Vries, 2010; Montomoli et al., 2012; Phillips, 2003). There is a trend where older epidemiological studies on MG tend to report the lower incidence and prevalence rates (Carr et al., 2010; Phillips, 2003), and increasing incidence rates over time have been shown in particular regarding late‐onset MG (Matsuda et al., 2005; Pakzad, Aziz, & Oger, 2011; Vincent, Clover, Buckley, Grimley Evans, & Rothwell, 2003). These increasing numbers may be partly explained by improved diagnostic techniques as well as emerging research‐driven knowledge about the disease. Improved treatment regimens as well as an aging population are other likely contributing factors. Nevertheless, true regional differences do exist, especially with regard to certain MG subgroups, for example, MG with antibodies against muscle‐specific tyrosine kinase (MuSK + MG), which is more common in Southern Europe than in Northern Europe (Niks, Kuks, & Verschuuren, 2007; Tsiamalos, Kordas, Kokla, Poulas, & Tzartos, 2009).

Accurate epidemiological information is important as it contributes to the understanding of disease mechanisms and provides a framework for planning the effective distribution of healthcare resources. Furthermore, knowledge of geographical variations is important when using results of MG studies from other countries in a clinical setting.

The epidemiology of MG in Sweden has traditionally been a sparsely studied subject, and reported prevalence rates vary markedly. Kalb et al reported MG prevalence to be 14.1 per 100,000 inhabitants in the county of Stockholm 1998 (Kalb, Matell, Pirskanen, & Lambe, 2002). Fang et al reported an MG prevalence of 24.8 per 100,000 inhabitants in Sweden 2010 (Fang et al., 2015), and we reported an MG prevalence rate of 19.9 per 100,000 inhabitants in Jönköping county 2014 (Westerberg, Landtblom, & Punga, 2018).

With improving disease knowledge, it could be argued that MG subgroups such as early‐onset MG (EOMG; onset < 50 years), late‐onset MG (LOMG; onset ≥ 50 years), juvenile onset MG (JOMG; onset < 18 years), and thymoma‐associated MG (TOMG), constitute different disease entities, which underlines the need to separate the epidemiology of the different subgroups. To date, such information is however almost completely absent.

We conducted a nationwide register‐based study of MG patients from the year 2006 to 2016, to document the current epidemiology of MG and MG subgroups in Sweden.

## 2 MATERIALS AND METHODS

### 2.1 Study design and ethical approval

This is a Swedish, nationwide register‐based cohort study approved by the Regional Ethical Review Board, Uppsala, Sweden (Dnr: 2017/279).

Data were acquired from the following four registers, provided by the National Board of Health and Welfare in Sweden (Socialstyrelsen):

1. The Swedish National Patient Register (NPR) was established in 1964 and contains patient information on public hospital admissions (e.g., dates of admission, medical diagnoses, surgical procedures), and from 2001 additionally information on outpatient visits to public and private caregivers, including visits to day surgery and psychiatric units. The inpatient part of the register covers all of Sweden from 1987 and reports 1%–2% of basic data missing during later years (Ludvigsson et al., 2011). The outpatient part of the register reported 4% of data missing in the year 2016 (Welfare, 2018). Primary care is not yet covered in the NPR.
2. The Swedish Prescribed Drug Register (SPDR) was established in 2005 and contains data with patient identities for all dispensed prescribed drugs to the entire Swedish population (Wettermark et al., 2007).
3. The Swedish Cause of Death Register (CDR) is available since 1961 and contains nationwide information about date of death and cause of death, as well as other relevant diagnoses contributing to the eventual death (Brooke et al., 2017).
4. The Swedish Cancer Registry (CR) was established in 1958 and claims to provide almost complete national coverage as it is mandatory for every healthcare provider to report newly detected cancer cases to the registry (Barlow, Westergren, Holmberg, & Talback, 2009).

### 2.2 Study population

Patients with (a) classification codes for MG; ICD‐9 code 358A or ICD‐10 code G70.0, in the NPR and/or (b) two or more prescriptions of pyridostigmine (ATC N07AA02) or ambenonium (ATC N07AA30) in SPDR were considered to have an MG diagnosis, in accordance with the MG identification method validated in a Swedish setting by Fang et al. (2015). Patients who were identified in the registers as having the diagnosis of MG at some point during the years 1986–2016, and were alive entering the year 2006, were included in the study cohort.

A total of 5,878 (3,191 women, 54.3%) patients were identified 1986–2018. Of these, 927 patients (481 women, 51.9%) had died before 2006 and 215 (95 women, 44.2%) patients were diagnosed after 2016 and therefore not included in the study. Four thousand seven hundred and thirty six patients (2,615 women, 55.2%) were included in the final study. Of them, 2,138 (1,310 women, 61.3%) received their first MG diagnosis date before 2006 and 2,598 (1,305 women, 50.2%) were diagnosed between 2006 and 2016. One thousand one hundred and twenty nine patients (563 women, 49.9%) died during the study period.

### 2.3 MG subgroups

Myasthenia Gravis onset was defined as the date of first MG diagnosis in the registers or the first date of prescription of pyridostigmine or ambenonium—whichever occurred first.

The MG cohort was divided into four subgroups:

1. EOMG: All patients with a disease onset at 18–49 years of age, without thymoma.
2. LOMG: All patients with a disease onset ≥50 years of age, without thymoma.
3. JOMG: All patients with a disease onset <18 years of age, without thymoma.
4. TOMG: All patients with a thymoma, as identified by ICD‐10 code D15.0, D38.4, or C37.9 in the NPR or in the CR registers.

### 2.4 Incidence and prevalence rates

Annual population statistics on age and gender was obtained for Sweden and the 25 counties from Statistics Sweden (https://www.scb.se/en/finding-statistics/statistics-by-subject-area/population/) for the years 2006–2016.

### 2.5 Statistical analysis

The crude incidence and prevalence rates were calculated using denominators derived from Statistics Sweden from the years 2006 to 2016. Incidence and prevalence rates were calculated per 100,000 inhabitants and 95% confidence intervals (95% CI) were calculated using Poisson distribution. Student's *t* test (for parametric data) and Mann–Whitney *U* test (for non‐parametric data) were used for analyzing continuous variables. A *p*‐value < .05 was considered significant. The statistical analyses were performed in GraphPad Prism version 6.0h for Mac (Graphpad software, www.graphpad.com).

## 3 RESULTS

### 3.1 Incidence

In total, there were 2,598 (1,305 women, 50.2%) new MG cases in Sweden 2006–2016. Of these, 588 [22.6%, 393 women (66.8%)] were EOMG, 1,804 [69.4%, 806 women (44.7%)] were LOMG, 120 [4.6%, 65 women (52.2%)] were TOMG, and 86 [3.3%, 41 women (47.7%)] were JOMG. Mean age of MG diagnosis for all MG patients (AllMG) was 56.0 ± 19.9 years, for EOMG 35.5 ± 8.9 years, for LOMG 68.3 ± 10.2 years, for TOMG 54.6 ± 16.6 years, and for JOMG 10.6 ± 5.0 years.

**Figure 1:**
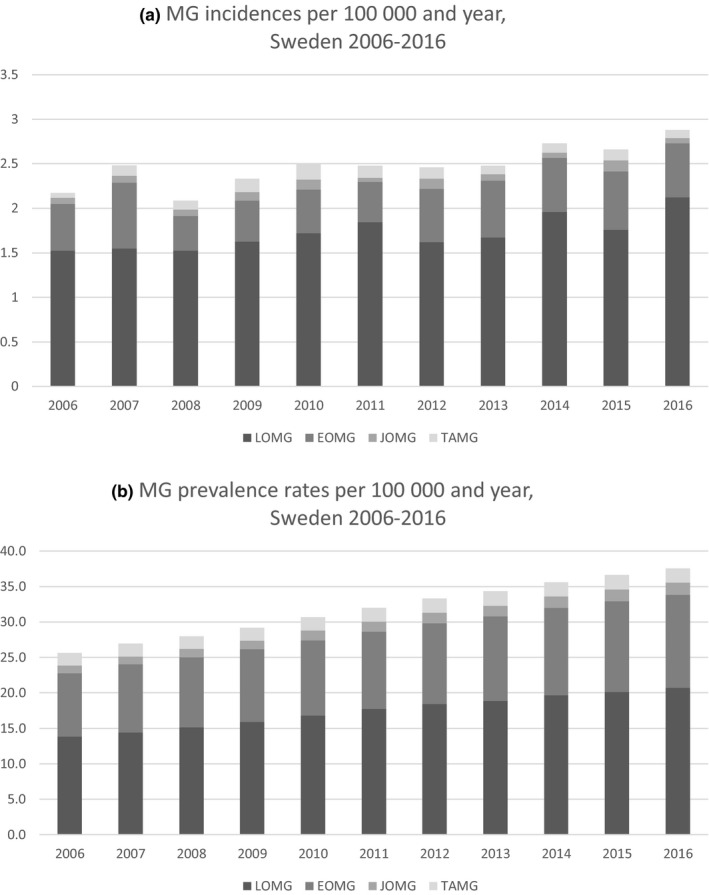
Incidence (a) and prevalence (b) rates of Myasthenia Gravis (MG) and MG subgroups in Sweden 2006–2016

As shown in Figure 1a, MG incidence rates in Sweden 2006–2016 varied between 2.1 and 2.9/100,000 per year (average 2.5/100,000), with a tendency toward higher incidence rates during the later years. Subgroup incidences (Table 1) varied from year to year with no consistent pattern over time, although there was a slight increase in LOMG incidence rates from 2006 to 2016.

**Table 1:** Incidence and prevalence rates of MG and MG subgroups in Sweden 2006–2016

	Incidence in cases/100,000 and year (95% CI)					Prevalence in cases/100,000 (95% CI)				
	ALL MG	JOMG	EOMG	LOMG	TAMG	ALL MG	JOMG	EOMG	LOMG	TAMG
2006										
All	2.2 (1.9–2.5)	0.1 (0.0–0.1)	0.5 (0.4–0.7)	1.5 (1.3–1.8)	0.1 (0.0–0.1)	24.7 (23.6–25.7)	1.1 (0.9–1.3)	8.9 (8.3–9.5)	13.0 (12.2–13.7)	1.7 (1.4–2.0)
F	2.2 (1.7–2.6)	0.0 (0.0–0.1)	0.7(0.5–0.9)	1.4 (1.1–1.8)	0.0 (0.0–0.1)	29.7 (28.1–31.3)	1.2 (0.9–1.5)	12.9 (11.9–14.0)	13.4 (12.3–14.5)	2.2 (1.8–2.6)
M	2.2 (1.7–2.6)	0.1 (0.0–0.2)	0.4 (0.2–0.5)	1.6 (1.3–2.0)	0.1 (0.0–0.2)	19.5 (18.3–20.8)	1.0 (0.7–1.2)	4.8 (4.2–5.5)	12.6 (11.5–13.6)	1.2 (0.9–1.5)
2007										
All	2.5 (2.2–2.8)	0.1 (0.0–0.1)	0.7 (0.6–0.9)	1.5 (1.3–1.8)	0.1 (0.0–0.2)	26.1 (25.1–27.1)	1.1 (0.9–1.3)	9.6 (8.9–10.2)	13.7 (13.0–14.5)	1.7 (1.4–2.0)
F	2.7 (2.3–3.2)	0.1 (0.0–0.2)	1.0 (0.7–1.3)	1.5 (1.1–1.8)	0.2 (0.1–0.3)	31.3 (29.7–32.9)	1.3 (0.9–1.6)	13.8 (12.8–14.9)	14.0 (12.9–15.1)	2.2 (1.8–2.6)
M	2.2 (1.8–2.7)	0.1 (0.0–0.1)	0.5 (0.3–0.7)	1.6 (1.3–2.0)	0.1 (0.0–0.1)	20.9 (19.5–22.2)	1.0 (0.7–1.3)	5.2 (4.6–5.9)	13.4 (12.3–14.5)	1.2 (0.9–1.5)
2008										
All	2.1 (1.8–2.4)	0.1 (0.0–0.1)	0.4 (0.3–0.5)	1.5 (1.3–1.8)	0.1 (0.0–0.2)	27.1 (26.0–28.2)	1.2 (0.9–1.4)	9.8 (9.2–10.5)	14.4 (13.6–15.2)	1.7 (1.4–1.9)
F	1.8 (1.4–2.2)	0.1 (0.0–0.1)	0.5 (0.3–0.7)	1.2 (0.9–1.5)	0.0 (0.0–0.1)	31.9 (30.3–33.6)	1.3 (1.0–1.6)	14.2 (13.1–15.3)	14.4 (13.3–15.4)	2.1 (1.7–2.5)
M	2.4 (1.9–2.8)	0.1 (0.0–0.2)	0.3 (0.1–0.5)	1.8 (1.4–2.2)	0.2 (0.0–0.3)	22.2 (20.9–23.6)	1.0 (0.7–1.3)	5.5 (4.8–6.1)	14.5 (13.4–15.6)	1.3 (1.0–1.6)
2009										
All	2.3 (2.0–2.6)	0.1 (0.0–0.2)	0.5 (0.3–0.6)	1.6 (1.4–1.9)	0.1 (0.1–0.2)	28.4 (27.3–29.5)	1.3 (1.0–1.5)	10.2 (9.5–10.8)	15.2 (14.4–16.0)	1.8 (1.5–2.0)
F	2.6 (2.1–3.0)	0.1 (0.0–0.2)	0.6 (0.4–0.8)	1.7 (1.4–2.1)	0.1 (0.0–0.2)	33.5 (31.9–35.2)	1.4 (1.0–1.7)	14.6 (13.5–15.7)	15.4 (14.2–16.5)	2.1 (1.7–2.5)
M	2.1 (1.7–2.5)	0.1 (0.0–0.2)	0.3 (0.1–0.5)	1.5 (1.2–1.9)	0.2 (0.1–0.3)	23.3 (21.9–24.6)	1.1 (0.8–1.4)	5.7 (5.0–6.3)	15.1 (14.0–16.2)	1.4 (1.0–1.7)
2010										
All	2.5 (2.2–2.8)	0.1 (0.0–0.2)	0.5 (0.3–0.6)	1.7 (1.5–2.0)	0.2 (0.1–0.3)	29.7 (28.6–30.8)	1.4 (1.1–1.6)	10.5 (9.9–11.2)	16.0 (15.2–16.8)	1.9 (1.6–2.1)
F	2.5 (2.1–3.0)	0.2 (0.1–0.3)	0.7 (0.4–0.9)	1.5 (1.2–1.9)	0.2 (0.1–0.3)	34.9 (33.2–36.6)	1.5 (1.2–1.9)	15.2 (14.0–16.3)	16.0 (14.8–17.1)	2.2 (1.8–2.7)
M	2.5 (2.0–2.9)	0.1 (0.0–0.1)	0.3 (0.2–0.5)	1.9 (1.5–2.3)	0.1 (0.0–0.3)	24.5 (23.1–26.0)	1.2 (0.9–1.5)	5.9 (5.2–6.6)	16.0 (14.9–17.2)	1.5 (1.1–1.8)
2011										
All	2.5 (2.2–2.8)	0.0 (0.0–0.1)	0.5 (0.3–0.6)	1.8 (1.6–2.1)	0.1 (0.1–0.2)	31.1 (30.0–32.2)	1.4 (1.1–1.6)	10.9 (10.2–11.5)	16.9 (16.1–17.8)	1.9 (1.7–2.2)
F	2.3 (1.9–2.7)	0.0 (0.0–0.1)	0.6 (0.4–0.8)	1.6 (1.2–1.9)	0.1 (0.0–0.2)	36.2 (34.5–37.9)	1.6 (1.2–1.9)	15.6 (14.5–16.7)	16.8 (15.6–18.0)	2.3 (1.8–2.7)
M	2.7 (2.2–3.1)	0.0 (0.0–0.1)	0.3 (0.2–0.5)	2.1 (1.7–2.6)	0.1 (0.0–0.3)	26.0 (24.5–27.4)	1.2 (0.9–1.5)	6.1 (5.4–6.8)	17.1 (15.9–18.3)	1.6 (1.2–1.9)
2012										
All	2.5 (2.1–2.8)	0.1 (0.0–0.2)	0.6 (0.4–0.8)	1.6 (1.4–1.9)	0.1 (0.1–0.2)	32.2 (31.0–33.3)	1.5 (1.2–1.7)	11.4 (10.7–12.0)	17.4 (16.5–18.2)	1.9 (1.7–2.2)
F	2.4 (2.0–2.8)	0.1 (0.0–0.2)	0.8 (0.5–1.0)	1.4 (1.0–1.7)	0.2 (0.1–0.3)	37.2 (35.5–38.9)	1.7 (1.3–2.0)	16.2 (15.1–17.3)	17.0 (15.8–18.1)	2.3 (1.8–2.7)
M	2.5 (2.1–3.0)	0.1 (0.0–0.2)	0.4 (0.2–0.6)	1.9 (1.5–2.3)	0.1 (0.0–0.2)	27.1 (25.7–28.6)	1.2 (0.9–1.6)	6.5 (5.8–7.2)	17.8 (16.6–19.0)	1.6 (1.2–1.9)
2013										
All	2.5 (2.2–2.8)	0.1 (0.0–0.1)	0.6 (0.5–0.8)	1.7 (1.4–1.9)	0.1 (0.0–0.2)	33.3 (32.1–34.4)	1.5 (1.3–1.8)	11.9 (11.2–12.6)	17.9 (17.1–18.7)	2.0 (1.7–2.3)
F	2.7 (2.2–3.2)	0.1 (0.0–0.2)	0.8 (0.6–1.1)	1.7 (1.3–2.0)	0.1 (0.0–0.3)	38.4 (36.7–40.2)	1.7 (1.4–2.1)	16.9 (15.7–18.1)	17.5 (16.3–18.6)	2.3 (1.9–2.8)
M	2.2 (1.8–2.7)	0.1 (0.0–0.1)	0.5 (0.3–0.6)	1.7 (1.3–2.0)	0.0 (0.0–0.1)	28.1 (26.6–29.6)	1.3 (1.0–1.6)	6.8 (6.1–7.6)	18.3 (17.1–19.6)	1.6 (1.2–2.0)
2014										
All	2.7 (2.4–3.1)	0.1 (0.0–0.1)	0.6 (0.5–0.8)	2.0 (1.7–2.2)	0.1 (0.0–0.2)	34.3 (33.2–35.5)	1.6 (1.3–1.8)	12.2 (11.6–12.9)	18.6 (17.7–19.4)	1.9 (1.7–2.2)
F	2.8 (2.3–3.3)	0.1 (0.0–0.1)	0.9 (0.6–1.1)	1.8 (1.4–2.1)	0.1 (0.0–0.2)	39.6 (37.8–41.3)	1.8 (1.4–2.2)	17.5 (16.3–18.6)	18.0 (16.8–19.2)	2.3 (1.9–2.7)
M	2.7 (2.2–3.1)	0.1 (0.0–0.1)	0.3 (0.2–0.5)	2.2 (1.7–2.6)	0.1 (0.0–0.2)	29.1 (27.5–30.6)	1.3 (1.0–1.7)	7.0 (6.3–7.8)	19.1 (17.9–20.3)	1.6 (1.2–2.0)
2015										
All	2.7 (2.3–3.0)	0.1 (0.1–0.2)	0.7 (0.5–0.8)	1.8 (1.5–2.0)	0.1 (0.1–0.2)	35.2 (34.0–36.4)	1.7 (1.4–1.9)	12.7 (12.0–13.4)	18.8 (18.0–19.7)	2.0 (1.7–2.2)
F	2.6 (2.2–3.1)	0.1 (0.0–0.2)	0.9 (0.6–1.2)	1.5 (1.1–1.8)	0.2 (0.1–0.3)	40.4 (38.7–42.2)	1.9 (1.5–2.3)	18.1 (16.9–19.3)	18.1 (16.9–19.3)	2.3 (1.9–2.8)
M	2.7 (2.2–3.1)	0.1 (0.0–0.2)	0.4 (0.2–0.6)	2.0 (1.6–2.4)	0.1 (0.0–0.1)	30.0 (28.4–31.5)	1.5 (1.1–1.8)	7.3 (6.6–8.1)	19.5 (18.3–20.8)	1.6 (1.2–2.0)
2016										
All	2.9 (2.5–3.2)	0.1 (0.0–0.1)	0.6 (0.5–0.8)	2.1 (1.8–2.4)	0.1 (0.0–0.1)	36.1 (34.9–37.3)	1.7 (1.4–1.9)	13.0 (12.3–13.7)	19.4 (18.6–20.3)	1.9 (1.6–2.2)
F	2.7 (2.2–3.1)	0.0 (0.0–0.1)	0.8 (0.6–1.1)	1.7 (1.4–2.1)	0.1 (0.0–0.2)	41.2 (39.4–43.0)	1.8 (1.5–2.2)	18.6 (17.4–19.8)	18.5 (17.3–19.7)	2.3 (1.9–2.7)
M	3.1 (2.6–3.6)	0.1 (0.0–0.2)	0.4 (0.2–0.5)	2.5 (2.1–3.0)	0.1 (0.0–0.2)	31.0 (29.5–32.5)	1.5 (1.2–1.9)	7.5 (6.7–8.2)	20.4 (19.2–21.7)	1.6 (1.2–1.9)

Abbreviations: CI, confidence interval; EOMG, early‐onset MG; JOMG, juvenile onset MG; LOMG, late‐onset MG; MG, Myasthenia Gravis; TAMG, thymoma‐associated MG.

### 3.2 Regional incidences

**Figure 2:**
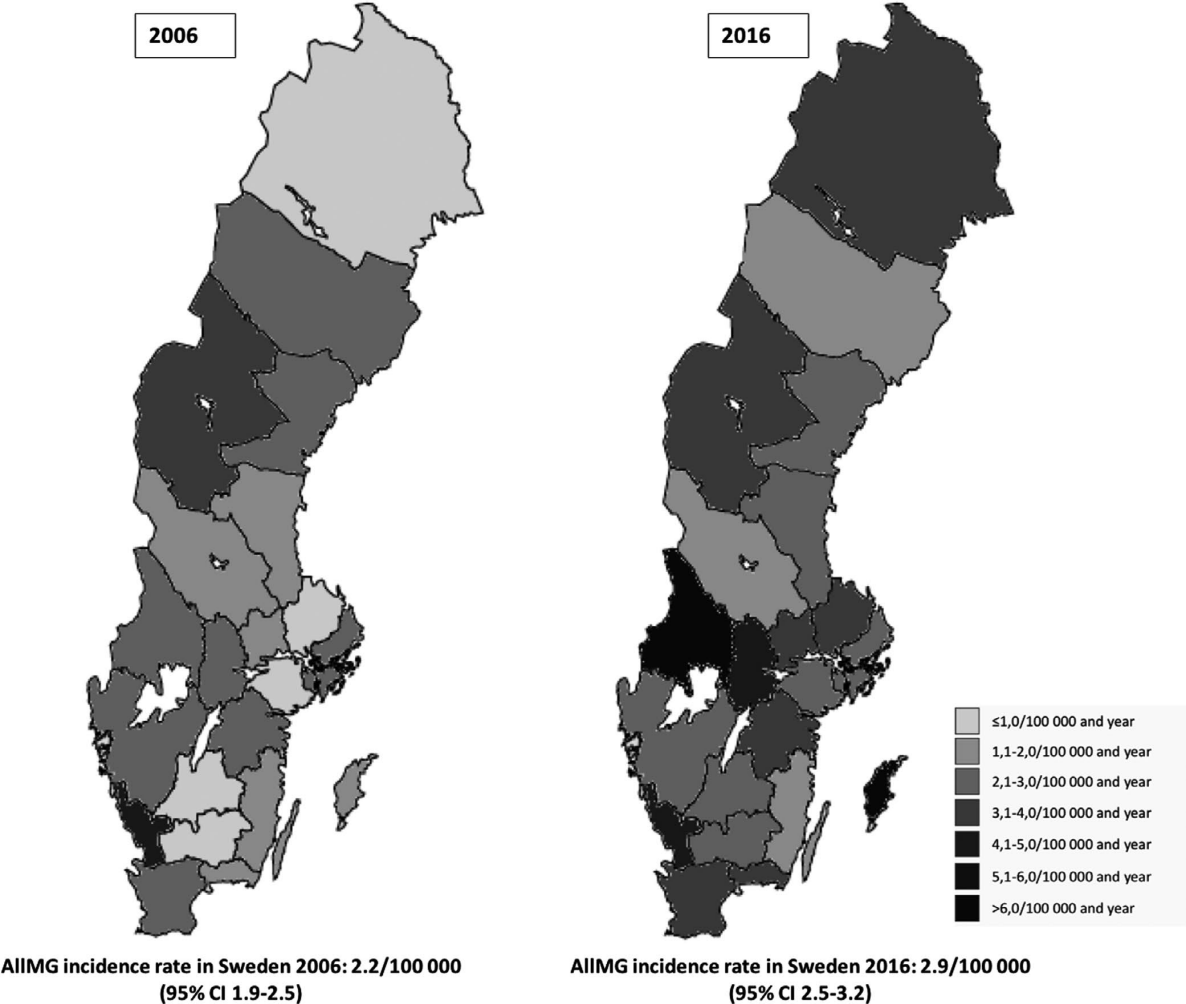
Myasthenia Gravis incidence rates in the 21 Swedish counties 2006 and 2016

There was no clear or consistent difference in incidence rates between different Swedish regions over the years, although there was an increase in Sweden as a whole from 2006 to 2016. When comparing the three largest regions (Malmö, Gothenburg, and Stockholm; each with inhabitants of more than one million), there were in general slightly higher incidence rates in the southernmost region (Malmö) than in the other two regions (Stockholm and Gothenburg), especially during later years. Incidence rates for all regions at the beginning and end of the study are shown in Figure 2.

### 3.3 Gender incidences

Gender incidence rates for all MG and subgroups are shown in Table 1. Female incidences were consistently higher in EOMG over the years. Male incidences were slightly higher in LOMG.

### 3.4 Prevalence

**Figure 3:**
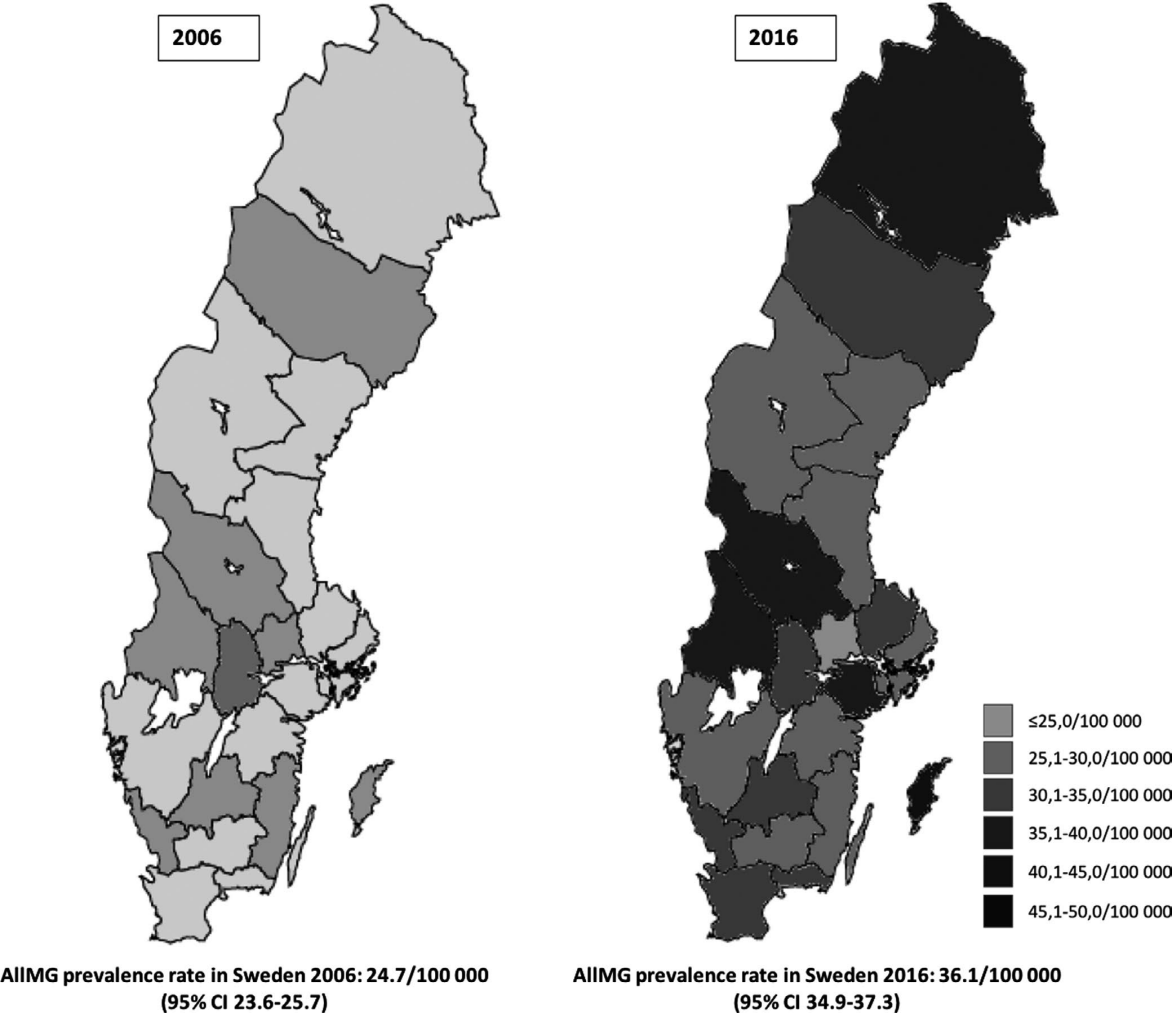
Myasthenia Gravis prevalence rates in the 21 Swedish counties 2006 and 2016

The prevalence of MG increased over time from 2006 to 2016 in Sweden as well as regionally within Sweden (Figure 3). The increased prevalence rates were noted in all subgroups except for TOMG. Prevalence increased for LOMG from 13.0/100,000 (95% CI: 12.2–13.7) in 2006 to 19.4/100,000 (95% CI: 18.6–20.3) in 2016 (*p* < .0001). EOMG prevalence increased from 8.9/100,000 (95% CI: 8.3–9.5) in 2006 to 13.0/100,000 (95% CI: 12.3–13.7) in 2016 (*p* < .0001). Prevalence rates for JOMG increased from 1.1/100,000 (95% CI: 0.9–1.3) in 2006 to 1.7/100,000 (95% CI: 1.4–1.9) in 2016 (*p* < .0001). For TOMG, the prevalence remained fairly stable (1.7–1.9, *p* = .3975) over the time period. Prevalence rates for all MG and MG subgroups are visualized in Figure 1b.

Of the 4,736 patients in the study, 1,357 (958 women, 70.6%) had EOMG, 2,923 (1,396 women, 47.8%) had LOMG, 279 (168 women, 60.2%) had TOMG, and 177 (93 women, 52.5%) had JOMG. The distribution of the subgroups in all Sweden was fairly stable over the years 2006–2016, with LOMG accounting for about 54% of MG diagnoses, EOMG for about 36%, JOMG for about 4%, and TOMG for 6%.

The average age of all MG patients in Sweden was stable at approximately 60 years over the years 2006–2016.

### 3.5 Regional prevalences

**Figure 4:**
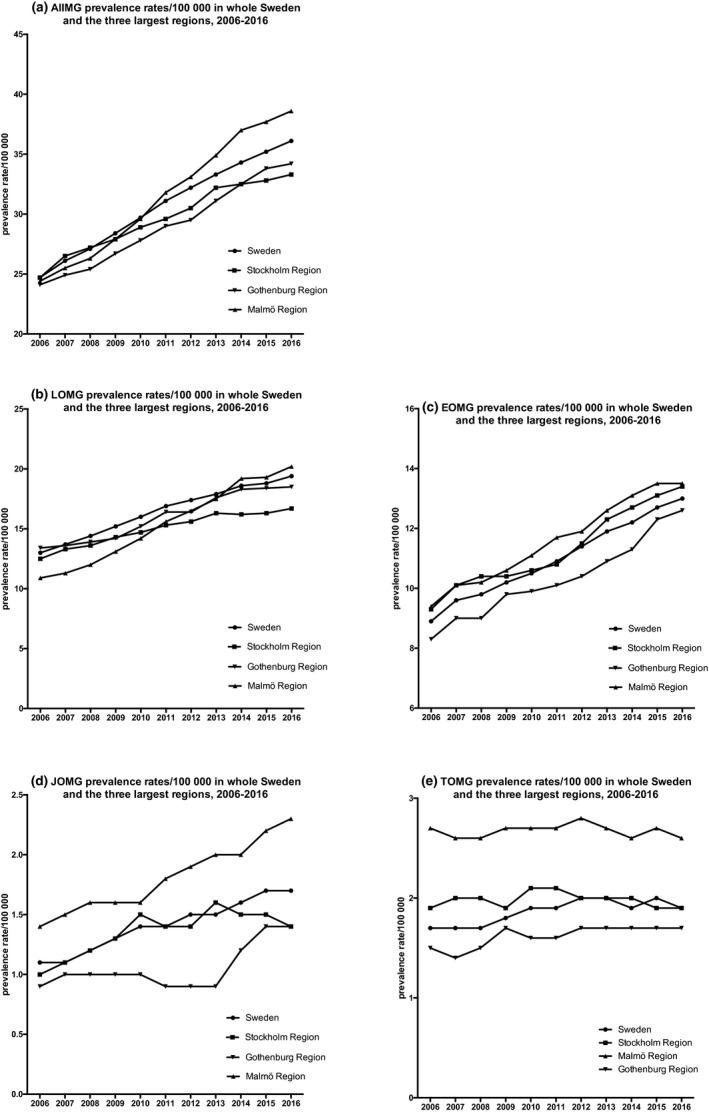
Prevalence rates of Myasthenia Gravis (MG, a) and MG subgroups (b–e) in all Sweden and the three largest regions (Stockholm, Malmö, and Gothenburg) 2006–2016

The prevalence rates differed between regions; however, as some regions are very small, a difference of one single patient could cause striking differences in prevalence, and a comparison between all regions would therefore not be meaningful. There was a difference in average prevalence rates between the three largest regions (with populations over one million) with higher AllMG, EOMG, JOMG, and TOMG prevalence rates in the Malmö region than in the Gothenburg and Stockholm regions and higher LOMG prevalence rates in the Gothenburg than in the Stockholm region. The prevalence rates in these regions 2006–2016 are shown in Figure 4a–e.

### 3.6 Gender prevalence

The female: male ratio for MG patients in Sweden was fairly stable over the years both for AllMG and in the subgroups, with the highest ratio of women in the EOMG subgroup (71%–73%). There was a slight tendency toward lower proportions of females with AllMG in 2016 (than in 2006); however, this pattern was not consistent within the regions.

**Figure 5:**
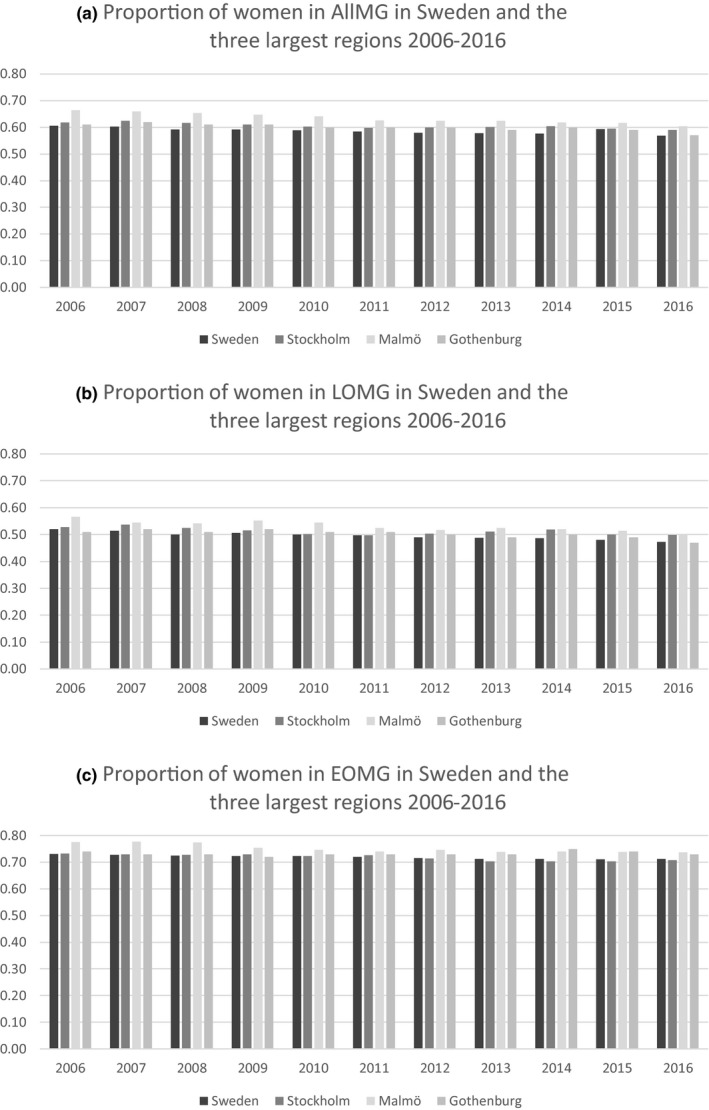
Proportion of women in Myasthenia Gravis (MG, a), as well as late‐onset MG (LOMG, b) and early‐onset MG (EOMG, c) in Sweden and the three largest regions (Stockholm, Malmö, and Gothenburg) 2006–2016

When comparing the three largest regions, there was in general a higher proportion of women with MG in these regions than in all Sweden, especially in the southernmost region (Malmö; Figure 5). Furthermore, there was a slightly higher proportion of women in both the LOMG and the EOMG subgroups in Malmö compared to Stockholm and Gothenburg.

## 4 DISCUSSION

This is the first nationwide study of MG incidence in Sweden. It is also one of few studies to document incidence and prevalence of different MG subgroups within the same population.

Fang et al. (2015) have previously reported the prevalence of MG in Sweden in 2010, but nationwide prevalence rates over time have not been previously described.

Here, we report a Swedish incidence of MG of 2.9 per 100,000 in 2016, which is a rate similar to what has been reported in other recent studies (Aragones et al., 2014; Breiner et al., 2016; Gattellari, Goumas, & Worthington, 2012; Lai & Tseng, 2010). Furthermore, we report a current nationwide crude prevalence of 36.1 per 100,000, which is among the higher prevalence rates reported, however still in agreement with some other studies (Aragones et al., 2017; Breiner et al., 2016). This prevalence is also much higher than the reported MG prevalence of 14.1 in 100,000 in Stockholm, Sweden in 1998. Accordingly, we found a significant increase in prevalence over the study period (2006–2016), which is in line with a reported rise in regional prevalence rates of MG over time (Breiner et al., 2016; Carr et al., 2010; Holtsema et al., 2000; Lai & Tseng, 2010; Pallaver et al., 2011). Different contributing causes to this increase have been suggested:

Improved MG care with MG patients now having similar life expectancies to the general population is encouraging; however, it has not been clearly identified what particular changes in MG care account for this. To our knowledge, the available treatment options for MG have not changed significantly in Sweden over the period studied and neither have the national treatment recommendations. One could speculate that improvements in intensive care can have played a role; however, we have clinically observed a general tendency to fewer MG‐related intensive care admissions in recent years. Furthermore, at least in Sweden, patients appear to be less often treated with intravenous corticosteroids in favor of oral tapering regimens in recent years. There are to date no studies which confirm these clinical observations and the causality is unclear.

An aging population is a common cause of increasing disease prevalence over time and even though the study period in relation to this could be considered relatively short, the life expectancy increased by 1.2 years for women and 1.9 years for men in Sweden between 2006 and 2016. This may play a considerable role in the increase in prevalence, at least in the LOMG group.

An increased disease awareness of MG among healthcare staff may result in more patients correctly being given the diagnosis, although it can also lead to a higher risk of over‐diagnosis. As the method to identify the patients was identical throughout the study and the total time‐span was only 11 years, it is doubtful that increased disease awareness had a serious impact. There was also no significant increase in incidence, which would be an expected first sign of an improved diagnostic set‐up.

Improved documentation and coverage by the national registers could lead to increasing prevalence figures over time; however, a parallel increase in incidence measures would similarly be expected here.

When the three largest health regions of Sweden were compared, we noted higher incidence and prevalence rates in Malmö, for all subgroups except LOMG. There are no previous descriptions of regional variations in MG within Sweden and the cause of the variations we saw in this study is unclear. Possibly, socioeconomic factors, as well as varying ethnic backgrounds of the populations within the regions, may play a role, but further evaluation is necessary.

The study has some limitations. As the register‐based methodology has the advantage of a supposed almost total nationwide coverage, there are disadvantages to the accuracy of the MG diagnosis. Despite using a validated MG identification method in this study (Fang et al., 2015), there is a risk of including individuals with a false diagnosis, as we did not have access to individual patient charts and therefore could not confirm that the diagnostic criteria of the Myasthenia Gravis Foundation of America were met. This might to some extent lead to an overestimation of the incidence and prevalence rates, while the increase in the rates over the years is probably reliable. Furthermore, we chose a pragmatic subgrouping method, limited to age at onset and the presence of a thymoma or not. It would have been desirable to consider antibody status and distribution of symptoms to obtain a more precise subgrouping; however, these data are unavailable in currently obtainable health registers.

As MG is uncommon, comparisons of smaller regions become statistically unreliable, which is why we chose to compare only the largest health regions. Another option would have been to cluster the smaller areas into larger ones for comparison. However, we chose to exclude them from the comparative analysis as we could not logically justify the clustering method.

Despite the above limitations, we believe that this study represents important and reliable information on Swedish MG epidemiology and that the reported rising prevalence rates described also in other countries indicate a need to improve awareness of MG among healthcare providers.

## 5 CONCLUSION

Over the time period of 2006–2016, incidence and prevalence rates of Myasthenia Gravis have increased in Sweden, and the prevalence rate of 36.1 per 100,000 in 2016 is high in comparison with many other countries. Yet, there seem to be regional differences within the country.

## CONFLICT OF INTEREST

The authors have no conflict of interest to declare.

## AUTHOR CONTRIBUTION

EW was responsible for conception and design of the study, for data acquisition, analysis and interpretation, and wrote the manuscript. ARP was responsible for conception and design of the study, for data analysis and interpretation and critically revised the manuscript. All authors reviewed and approved the final version of the manuscript.

### Peer Review

The peer review history for this article is available at https://publons.com/publon/10.1002/brb3.1819.

## Data Availability

The datasets analyzed during the current study are not publicly available due to the GDPR legislation but are available from the corresponding author on reasonable request.
